# Improving the prediction accuracy in classification using the combined data sets by ranks of gene expressions

**DOI:** 10.1186/1471-2105-9-283

**Published:** 2008-06-16

**Authors:** Ki-Yeol Kim, Dong Hyuk Ki, Hei-Cheul Jeung, Hyun Cheol Chung, Sun Young Rha

**Affiliations:** 1 Oral Cancer Research Institute, Yonsei University College of Dentistry, Seoul, 120-752, South Korea; 2 Cancer Metastasis Research Center, Yonsei University College of Medicine, Seoul, 120-752, South Korea; 3 National Biochip Research Center, Seoul, 120-752, South Korea; 4 Brain Korea 21 Project for Medical Science, Yonsei University College of Medicine, Seoul, 120-752, South Korea; 5 Yonsei Cancer Center, Yonsei University College of Medicine, Seoul, 120-752, South Korea; 6 Department of Internal Medicine, Yonsei University College of Medicine, Seoul, 120-752, South Korea

## Abstract

**Background:**

The information from different data sets experimented under different conditions may be inconsistent even though they are performed with the same research objectives. More than that, even when the data sets were generated from the same platform, the data agreement may be affected by the technical variation among the laboratories. In this case, it is necessary to use the combined data set after adjusting the differences between such data sets, for detecting the more reliable information.

**Results:**

The proposed method combines data sets posterior to the discretization of data sets based on the ranks of the gene expression ratios, and the statistical method is applied to the combined data set for predictive gene selection. The efficiency of the proposed method was evaluated using five colon cancer related data sets, which were experimented using cDNA microarrays with different RNA sources, and one experiment utilized oligonucleotide arrays. NCI-60 cell lines data sets were used, which were performed with two different platforms of cDNA microarrays and Affymetrix HU6800 oligonucleotide arrays. The combined data set by the proposed method predicted the test data sets more accurately than the separated data sets did. The biological significant genes were detected from the combined data set, which were missed on the separated data sets.

**Conclusion:**

By transforming gene expressions using ranks, the proposed method is not influenced by systematic bias among chips and normalization method. The method may be especially more useful to find predictive genes from data sets which have different scale in gene expressions.

## Background

Data sets that are created for the same purpose in different laboratories have accumulated rapidly. The results are often inconsistent due to the utilization of different platforms, sample preparations, or various technical variations. In this case, if a combined data set were analyzed after adjusting systematic biases that exist among different data sets derived from different experimental conditions, the power of statistical tests would be improved by an increase in the sample size.

When the results from different data sets are inconsistent, usually the common portions can be adapted for stability. This type of meta-analysis involves a set of classical statistical techniques [1] and has been applied to microarray data sets [2,3]. As a method to combine data sets, Lee et al. [4] simply standardized gene expression ratios of human and mouse microarray data sets and combined these two data sets for comparative functional genomics. To analyze a combined data set of two different data sets, the transformation of gene expression was introduced [5]. This method transforms the gene expression ratios of two data sets in the form of a reference experiment and the reference experiment is created as a mean vector for all experiments. Consequently, this method does not consider the difference in gene expression patterns that exists between different experimental groups. To account for the variability that resulted from the various confounding factors such as different experimental conditions, an ANOVA (Analysis Of Variance) model was introduced [6]. It is a flexible method for considering gene expression ratios and other clinical variables together, although it does not create a combined large data set for applying various analytical methods.

Sometimes gene expression ratios may include outliers as a result of incomplete experimental conditions, and these values can cause unreliable results by their strong influence. The usage of the categorized values of gene expression ratios can reduce the influence of outliers in this case and may improve the prediction accuracies in the classification of different experimental classes. The usage of the discrete values has advantage that is more concise to represent and specify, easier to use, and conducive to improved predictive accuracy [7]. The discretization of gene expression levels has been achieved [8].

The simplest discretization methods are the Equal Interval Width and Equal Frequency Intervals methods. Kerber [9] suggested the ChiMerge method and this method begins by placing each observed value into its own interval and proceeds by using the χ^2 ^test to determine when adjacent intervals should be merged. A number of entropy-based methods have recently come to the forefront of work on discretization [10]. Fayyad and Irani [10] use a recursive entropy minimization heuristic for discretization and couple this method with the Minimum Description Length criterion [11] to control the number of intervals produced over the continuous space. In addition, a nonparametric scoring method was applied to gene expression data to discretize gene expression ratios [12], which usually transforms expression ratios based on their ranks by each experiment. In this case, some genes are included in the same rank and the score can be calculated differently according to the order of ranks with same values, which requires more time to score as the number of samples increases.

In this study, gene expression ratios were transformed with their ranks for each data set. Next, the transformed data sets were combined and a nonparametric statistical method was applied to the combined data set to detect informative genes with high prediction accuracy. The performance of the proposed method using data sets derived from different platforms and different RNA sources was evaluated.

## Results

### A. The necessity of combining data sets

The relationship between the number of genes and OOB (Out of Bag) error rates was investigated using data A, data B and data AB, which represent the data sets with total RNA, amplified RNA, and the combined data, respectively.

The OOB error rates were calculated for randomly selected genes 500 times repeatedly with the same size and averaged them. The OOB error rates decreased as the number of informative genes increased.

**Figure 1:**
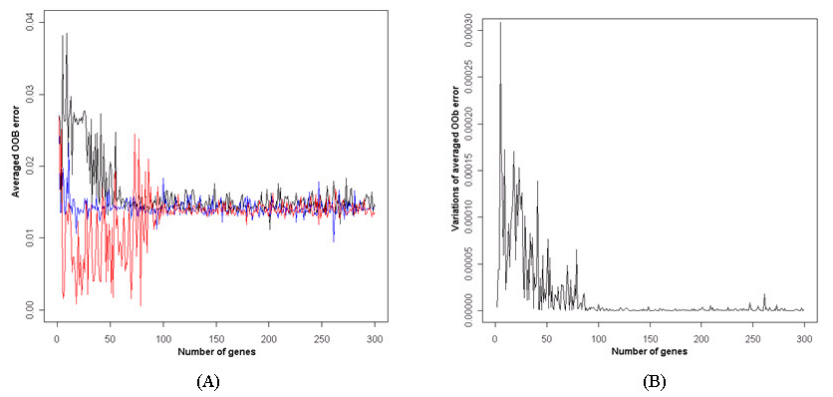
**Relationship of OOB error rates to the number of significant genes.** (A) Red: data A, black: data B, blue: data AB, which is a combined data set by the proposed method. (B) Variations of averaged OOB error rates among three different data sets, data A, data B, and data AB.

As shown in Figure 1A, the OOB error rates were decreased with a small number of genes when informative genes were used by their significance in discrimination. While there was a large variation in OOB error rates in the separated data sets, it was even more stable in the combined data set. Therefore, it was confirmed that the more stable discriminative gene set can be detected from the combined data set. In this case, that the OOB error rates had large variations as the number of informative genes was increased can be attributed to the addition of redundant genes. In data mining processes, it is generally known that the exclusion of redundant variables improves the power in discrimination [13]. Therefore, both significance and redundancy should be considered to detect the most discriminative gene set. Figure 1B showed that the variations among the three averaged OOB error rates were stable at 80–90 informative genes. This indicates that two separated data sets and a combined data set have almost the same power in discrimination with 80–90 informative genes. However, OOB error rates in a combined data set were stable with about 20 genes (Figure 1A).

### B. Improvement of prediction accuracy using combined data sets by the proposed method

The prediction accuracies were compared using two original colon cancer data sets as training data sets, which were experimented with different RNA sources.

**Figure 2:**
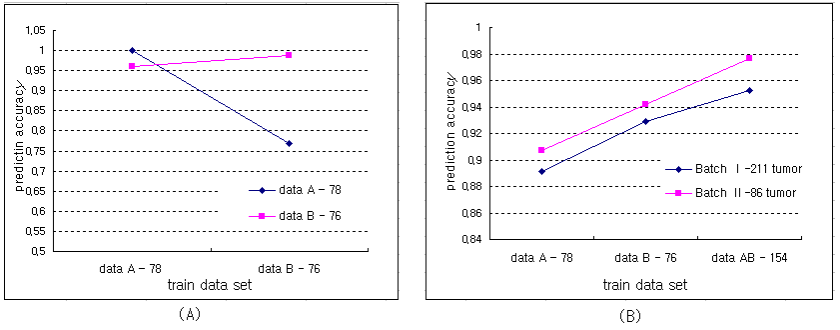
**Comparison of the prediction accuracies.** The number by the name of each data set represents the sample size of the data set. Seven significant genes with high prediction accuracy were used.

While the prediction accuracy of data B using data A as a train data set was higher than 95%, the accuracy of data A using data B was lower than 80% (Figure 2A). This indicated that the data set created using total RNA predicted the data set using amplified RNA more correctly. Figure 2B shows that the prediction accuracies of the two test data sets, Tumor 211 and Tumor 86. The prediction accuracy of the combined data set was higher than the separated data sets on two test data sets. Also, data B predicted test data sets with higher accuracy than data A, it could be caused that the two test data sets were also experimented using amplified RNA. The prediction accuracy was higher in Batch II-86 tumor than in Batch I-211 tumor data set.

### C. Comparison of the prediction accuracy with the Minimal Entropy (ME) method

**Figure 3:**
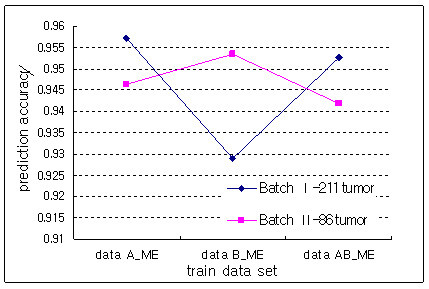
Comparison of the prediction accuracies in ME method.

Figure 3 shows the prediction accuracies of the separated and combined data sets transformed by the Minimal Entropy (ME) method.

Tumor 211 and Tumor 86 data sets were predicted more accurately with train data A and data B, respectively. This indicated that the prediction accuracy depends on train data sets in the ME method. Figure 3 also showed that there was not any improvement in prediction accuracy when a combined data set by ME method was used as train data set.

**Figure 4:**
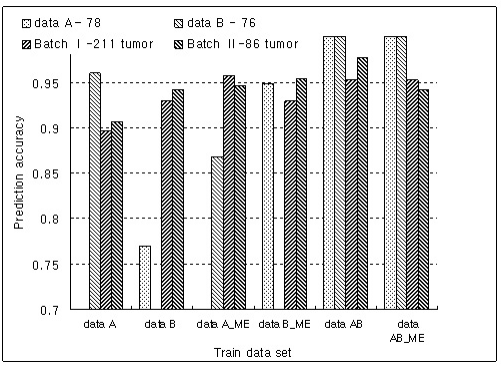
Comparison of the prediction accuracies between the proposed method and the ME method.

Figure 4 shows the comparison of prediction accuracies between the proposed method and ME method. While the combined data set by the ME method did not show any improvement in prediction accuracy compared with separated data sets, the proposed method improved it by combining data sets. The two combined data sets predicted the test data sets with high accuracy and the proposed method showed higher accuracies and smaller variations in accuracies on test data sets than did the ME method

### D. Description of significant genes selected from a combined data set by the proposed method

The descriptions of six discriminative genes selected from the combined data set, not two separated data sets, are summarized in Table 1.

**Table 1:** Description of six informative genes (one is duplicated among seven genes) selected from the combined data set after transformation by the proposed method.

**Gene ID**	**Gene name**	**UniGene ID**	**Symbol**	**Chromosomal Location**
AA485151	heat shock 105 kda/110 kda protein 1	Hs.36927	HSPH1	13q12.3
AA425217	cadherin 3, type 1, p-cadherin (placental)	Hs.554598	CDH3	16q22.1
AA464731	s100 calcium binding protein a11 (calgizzarin)	Hs.417004	S100A11	1q21
AA504130	cytoskeleton associated protein 2	Hs.444028	CKAP2	13q14
AA455925	four and a half lim domains 1	Hs.435369	FHL1	Xq26
AW050510	pyrroline-5-carboxylate reductase 1	Hs.458332	PYCR1	17q25.3

AA485151 was upregulated by over five-fold in colorectal adenocarcinoma [14]. AA425217 was published as a significant gene in colorectal cancer [15], and 16q22.1, where AA425217 is located, is a region that includes CDH1, which encodes cell-cell adhesion protein and is expressed in gastric cancer and lobular breast cancer. AA464731 is known to be a downregulated gene in the SW620 cell line [16], a metastatic colorectal cancer cell line. It is also significantly overexpressed in pancreatic cell lines [17]. AA504130 is located on 13q12.3, similarly to BRCA2, which is known as a marker of breast and ovarian cancer. The mutated gene for retinoblastoma is located on chromosome 13q14 [18], on which AA504130 is also located.

AA455925 is known as a E2F-1 regulated gene [19]. Xq26 is a region of two common chromosomal deletion regions, Xq25 and Xq26 [20], and is known to contribute to the malignant progression of gastric epithelial progenitor (GEP) endocrine carcinomas. Colorectal cancer is thought to be more common in men than in women. Xq26 is known as one of regions that contained multiple gains-of-function that were significantly more common in males than in females [21]. Since AW050510 is located at 17q25.3 and BIRC5 is at 17q25 and is known as a 'survivin expression colorectal cancer' [22,23], AW050510 is also expected to have similar characteristic to BIRC5.

The descriptions of 4 colorectal cancer related genes and 9 cancer related genes are summarized in Table 2. These genes were selected from a combined data set.

**Table 2:** Summarization of cancer related genes selected from a combined data set .

**Gene ID**	**Gene name**	**UniGene ID**	**Symbol**	**Chromosomal Location**
Colon cancer related genes				
N53057	chk1 checkpoint homolog (s. pombe)	Hs.24529	CHEK1	11q24-q24
R19158	aurora kinase a	Hs.250822	AURKA	20q13.2-q13.3
AA973748	fibrinogen silencer binding protein	Hs.30561	RAD54B	8q21.3-q22, 8q22.1
AA446462	bub1 budding uninhibited by benzimidazoles 1 homolog (yeast)	Hs.469649	BUB1	2q14
Cancer related genes				
N71159	metastasis associated 1	Hs.525629	MTA1	14q32.3
AA913127	glucosaminyl (n-acetyl) transferase 2, i-branching enzyme (i blood group)	Hs.519884	GCNT2	6p24
N53057	chk1 checkpoint homolog (s. pombe)	Hs.24529	CHEK1	11q24-q24
AA664219	nuclear receptor subfamily 3, group c, member 1 (glucocorticoid receptor)	Hs.122926	NR3C1	5q31.3
AI337292	ttk protein kinase	Hs.169840	TTK	6q13-q21
R19158	aurora kinase a	Hs.250822	AURKA	20q13.2-q13.3
AA12698	myosin, heavy polypeptide 11, smooth muscle	Hs.460109	MYH11	16p13.13-p13.12
AA446462	bub1 budding uninhibited by benzimidazoles 1 homolog (yeast)	Hs.469649	BUB1	2q14
AA453176	ataxia telangiectasia and rad3 related	Hs.271791	ATR	3q22-q24

### E. Improvement of prediction accuracies by combining data sets performed using different platforms

The prediction accuracies of combined data sets derived from different platforms were investigated.

**Figure 5:**
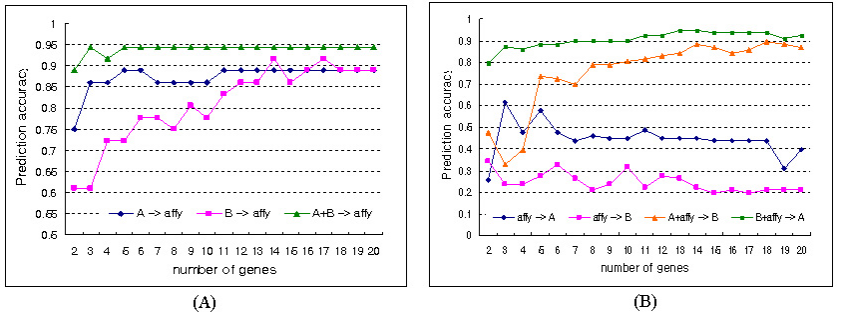
Comparison of prediction accuracies of single and combined data sets.

While the prediction accuracies of data A and data B on affy were low with a small number of genes, it increased as the number of genes was increased. By combining data A and data B, the prediction accuracy on affy was improved, as shown in Figure 5A. When affymetrix data was used as a train data set, its prediction accuracies on data A and data B were lower than 60%. However, after combining with data A or data B, it improved to higher than 90% (Figure 5B).

### F. Comparison of prediction accuracies of the proposed method in different platforms

The proposed method was evaluated using a NCI 60 cell line data set, and the prediction accuracies of the proposed method and the ME method were compared.

**Figure 6:**
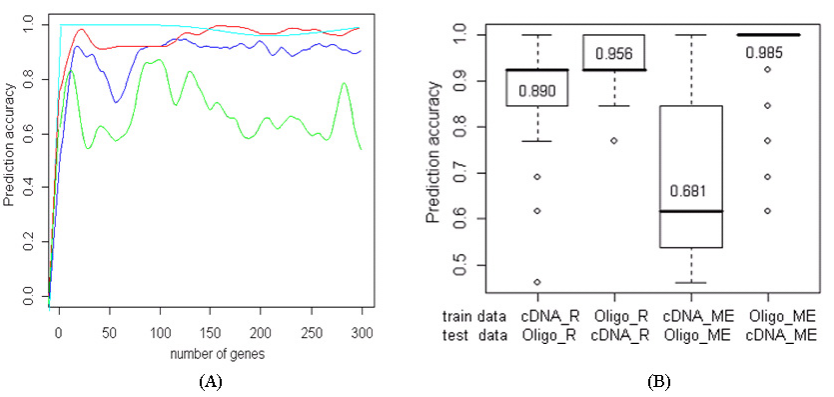
**Comparison of prediction accuracies under conditions of two test data sets and the number of informative genes.** (A) Blue: test Oligo data with cDNA by proposed method; Red: test cDNA data with Oligo data by proposed method; Green: test Oligo data with cDNA by ME method; Cyan: test cDNA data with Oligo data by ME method. (B) Summary of prediction accuracies using a boxplot. cDNA_R and Oligo_R are data sets transformed by the proposed method. cDNA_ME and Oligo_ME are data sets transformed by the ME method.

The prediction accuracies of both methods were compared as the number of genes was increased to 300, and they improved as the number of genes was increased, regardless of train and test data sets (Figure 6A). The prediction accuracies in the data sets that were transformed by the ME method were greatly different according to the train sets. The red and blue lines of Figure 6A displayed more stable fluctuation in the prediction accuracies than the green line, and they also showed similar patterns in prediction accuracies.

When Oligo data was used for train, the variation in accuracies was relatively small and prediction accuracy was high. It indicated that the Oligo data set predicted the cDNA data set more stably and accurately than the cDNA data set did. There was rare improvement in prediction after 20 or 30 genes, and this showed that a small informative gene set is sufficient for discrimination. It was also confirmed that the prediction accuracies were robust against the train data sets in the proposed method, while those in the ME method depended on the train data sets and there was significant difference between them (Figure 6B).

## Discussion

The designed 25-mer oligochips from Affymetrix provide an absolute value of expression in an RNA sample, while cDNA microarrays perform a two-color competitive hybridization that gives the transcript expression in two samples. Also, long oligonucleotide platforms (typically 60 to 80-mers) also use hybridization, the relative measurements resulted in higher precision than did absolute measurements on this platform [3]. Therefore, some experimental biases can exist as a result of the differences in the usage of absolute measurements and ratios.

Additionally, some previous studies indicated that the data sets from different microarray platforms should not be combined straightforwardly [24-27]. However, even when the data sets were generated from the same platform, the lab effect, especially when compounded with the RNA sample effect, plays a bigger role than the platform effect on data agreement [28].

There also exist inter-study biases among several microarray data sets tested with different RNA sources even when they are from the same laboratory and platform. Previous studies showed that there were some differences in results from data sets tested with different RNA sources, and the sensitivity to detect differential gene expression from a microarray data set using amplified RNA was also different compared to using total RNA [29,30].

An attempt to combine these different types of data sets is the usage of abstraction of expression values such as ranks or discretized values [9-12]. These methods reduce the variability in expression values from different microarray data sets. While there may be a slight loss of information by discretization, it is robust against outliers and fast and simple to understand.

In colon cancer data sets derived from cDNA microarrays, a data set created with total RNA predicted more accurately a data set created using amplified RNA than vice versa (Figure 2A). However, the data set, which was experimented upon using amplified RNA, showed better performance in the prediction of two test data sets than did a data set from total RNA (Figure 2B). It can be interpreted that the usage of same source can improve the prediction power. Also, the combined data set predicted two test data sets more accurately than did the separated data sets. With the ME method, there existed some interaction between test and train data sets even though it preserved high prediction accuracies on test data sets (Figure 3). This indicated that its prediction accuracy depended on test data sets and was not stable. Consequently, a combined data set by the proposed method showed the best performance in the prediction of test data sets (Figure 4). The top six discriminative genes selected from a combined data set, which were not detected from two separated data sets, were proven as genes associated with colon cancer by previous studies. Therefore, we believe that the usage of a combined data set is more reliable to detect biologically significant genes compared with separated data sets.

In the colon cancer data set derived from oligonucleotide arrays, the prediction accuracies were improved by combination with cDNA data sets. Although two data sets derived from different experimental conditions have different scales in gene expressions, such a different scale of gene expressions could be compensated by discretizing gene expression. Therefore, no transformation method was required to match these two types of data sets except only the ranking of gene expressions.

In the NCI 60 cell line data sets from two different platforms, different types of two data sets were used by alternating train and test data sets. The prediction accuracies in datasets that were transformed by the ME method were greatly different according to train sets, while those by the proposed method were accompanied by stable fluctuation in the prediction accuracies. The Oligo data set predicted the cDNA data set more stably and accurately than vice versa. While the prediction accuracies in the ME method depended on train and test data sets and the significant difference existing between them, they were more robust against train data in the proposed method (Figure 6B). In spite of the increase in the number of genes, the prediction power was not improved after 20 or 30 genes, and this indicated that small significant gene set is sufficient to predict different experimental groups.

In this study, we transformed microarray data using ranks of gene expressions to combine data sets created in different experimental conditions. The proposed method may be especially more useful to find discriminative genes from data sets that have different scales of gene expression ratios.

## Methods

### Data set

The data sets used in this study are summarized in Table 3.

**Table 3:** Colon cancer data sets for train and test (genes with missing entries were excluded).

**Data name**	**Experimental sources**	**# of genes**	**# of total samples**	**Normal group**	**Tumor group**
***Train data sets ***^***a***^					
Data A	Total RNA	12319	78	35	43
Data B	Amplified RNA	12319	76	37	39
Data AB	Combined data set	12319	154	72	82
***Test data sets ***^***a***^					
Tumor 86	Amplified RNA (Batch I)	17104	86	0	86
Tumor 211	Amplified RNA (Batch II)	17104	211	0	211
***Train and Test data set (Notterman et al., 2001)***					
Affy	Affymetrix HU6800	7464	36	18	18

^a^: Cancer Metastasis Research Center of Yonsei University, Seoul, Korea.

Two cDNA microarray data sets, data A and data B, experimented with 154 colorectal tissues (82 tumor and 72 normal) were used as train data sets for evaluation of the proposed method. These two cDNA microarray data sets derive from different RNA sources, which were total RNA and amplified RNA. Previous studies have concluded that there were differences between the results from these two types of data sets and the sensitivity to detect differential gene expression from microarray data sets using amplified RNA was also different compared to using total RNA [29,30]. It was also confirmed that systematic biases existed between these two data sets using unsupervised hierarchical cluster analysis [31].

Two more cDNA data sets, Tumor 86 and Tumor 211, were experimented with amplified RNA and under different batches, and they were used as test data sets. They included only colon tumor tissues. These colon cancer data sets performed with cDNA microarrays were from the Cancer Metastasis Research Center of Yonsei University, Seoul, Korea. One more colon cancer data set was used, which was performed with the Human 6800 Gene Chip Set (Affymetrix). It was obtained from microarray database of Princeton University [32] and it included experiments with the adenomas and their paired normal tissue [33].

To evaluate the performance of the proposed method in different platforms, NCI 60 cell line data sets derived from different platforms were also used. Gene expression data sets for NCI-60 using 9,706 cloned cDNA microarrays and 6,810 gene Affymetrix HU6800 oligonucleotide arrays were obtained separately from the additional files of Lee et al. [25], and the common 2,344 UniGene clusters were used for this study. Ovarian and colon cancer cell lines were used for this study among nine tumor cell lines, and these two groups included six and seven replications.

### Transformation method of gene expression ratios

#### Data preprocessing

Gene expression ratios were normalized such that they would have similar distributions across a series of arrays and the normalization process was executed using the 'limma' library of the R package [34]. The cDNA data in the NCI 60 cell line data sets included missing entries, and these were estimated by using the SeqKnn (Sequential k nearest neighbor) imputation method [35] before analysis.

#### Discretization by proposed method using rank of gene expression

For transformation of the data set, gene expression ratios are rearranged in order of expression ratios by each gene, and the ranks are matched with the corresponding experimental group. If the experimental groups are homogenous, the ranks within the same experimental group would be neighboring. This process can be seen as similar to the first step in the nonparametric Mann-Whitney U test. The process of discretization of gene expressions is summarized in the following steps:

(1) Rank the gene expression ratios within a gene for each data set.

(2) List in order of the ranks and assign the order of gene expressions to the corresponding experimental groups.

(3) Summarize the result of (2) in the form of a contingency table for each gene.

(4) Test the relationship between the gene expression patterns and experimental groups for each gene.

When there are three data sets to be combined, the data sets can be added by each entry as shown in Table 4 after the transformation of each data set by rank.

**Table 4:** Combination of contingency tables for three data sets (*t*_*ij *_= *a*_*ij *_+ *b*_*ij *_+ *c*_*ij*_)

	P1	P2	P3			P1	P2	P3			P1	P2	P3			P1	P2	P3
E1	a_11_	a_12_	a_13_		E1	b_11_	b_12_	b_13_		E1	c_11_	c_12_	c_13_		E1	t_11_	t_12_	t_13_
E2	a_21_	a_22_	a_23_	+	E2	b_21_	b_22_	b_23_	+	E2	c_21_	c_22_	c_23_	=	E2	t_21_	t_22_	t_23_
E3	a_31_	a_32_	a_33_		E3	b_31_	b_32_	b_33_		E3	c_31_	c_32_	c_33_		E3	t_31_	t_32_	t_33_
data set A					data set B					data set C					combined data set			

P1, P2 and P3 represent the three different phenotypes. E1, E2 and E3 represent three groups by ranks of gene expressions. a_ij_, b_ij _and c_ij _are the numbers of experiments belong to P_j _and E_i _at the same time in data A, data B and data C, respectively.

#### Discretization of expression ratios using recursive minimal entropy

A method for discretizing continuous attributes based on a minimal entropy (ME) heuristic, presented by Catlett [36] and Fayyad and Irani [10], was compared with the proposed method in the experimental study. The algorithm uses the class information entropy of candidate partitions to select binary boundaries for discretization. If there is a given set of instances S, a feature A, and a partition boundary T, the class information entropy of the partition induced by T, denoted E(A, T, S) is given by:

$$E(A,T;S)=\frac{\left|S_{1}\right|}{\left|S\right|}Ent(S_{1})+\frac{\left|S_{2}\right|}{\left|S\right|}Ent(S_{2})$$

For a given feature A, the boundary *T*_*min*_, which minimizes the entropy function over all possible partition boundaries, is selected as a binary discretization boundary. This method can be applied recursively to both of the partitions induced by *T*_*min *_until some stopping condition is achieved, thus creating multiple intervals on feature *A*. It must be evaluated *N-1 *times for each attribute with *N *the number of attribute values [37]. The library 'dprep' in R [34] was used for this method.

#### Nonparametric method for significant gene selection

After the summarization of gene expression ratios in the form of a contingency table for each gene, as shown in Table 5, a nonparametric statistical method was applied to the data sets for independency testing between gene expression patterns and experimental groups. The test statistics are calculated as follows for each gene:

**Table 5:** Summarization of discretized data using ranks of gene expressons.

		Experimental groups by phenotypes			
		
		P1	P2	P3	Marginal sum
Experimental group by rank (or ME^a^) of gene expression	E1	n_11_	n_12_	n_13_	r_1_
	E2	n_21_	n_22_	n_23_	r_2_
	E3	n_31_	n_32_	n_33_	r_3_
Marginal sum		c_1_	c_2_	c_3_	n

The significant genes can be selected by independency test between the phenotypes and gene expressions using this type of summarized data set. *c*_*i*_,*r*_*i *_represent the marginal sums of the *i*^*th *^column and row, respectively. *n *represents total number of experiments. **^a^: minimal entropy method**

$$\begin{array}{cc} \chi^{2}=\sum \frac{{\left[n_{ij}-\hat{E}(n_{ij})\right]}^{2}}{\hat{E}(n_{ij})}, & \hat{E}(n_{ij})=\frac{r_{i}c_{j}}{n} \end{array}$$

When the sample size for each experiment is small, generally less than five, Fisher's exact test is recommended rather than the Chi-square test.

### Classification method to evaluate the informative gene set selected from the combined data set

In order to evaluate the predictive accuracy of the selected significant gene set, the Random Forest (RF) test [38] was used to enable re-sampling while allowing repetition. The RF program in the R package [34] was used and it works using the following steps:

(1) Generate n data sets of bootstrap samples {B_1_, B_2_, ..., B_n_} by allowing repetition. (2) Use a B_k _to build a tree classifier T_k_, and classify B_m_s (m≠k) data (out-of-bag (OOB) samples). (3) Calculate classification errors of B_m_s and obtain an average for them which is the overall classification error (OOB error). (4) Calculate the prediction accuracy of test data sets using the classifier built in (2).

## Abbreviations

ANOVA: Analysis Of Variance; OOB error: Out Of Bag error; ME: Minimal Entropy.

## Authors' contributions

KYK participated in the design of algorithms, performed statistical analysis and drafted the manuscript. DHK performed the microarray experiments. HCJ participated in getting the consent form the patients and obtained the clinical data. HCC participated in the study design and data interpretation. SYR conceived of the study, participated in its design and coordination, and finalized manuscript.
